# *In vivo *development of dendritic orientation in wild-type and mislocalized retinal ganglion cells

**DOI:** 10.1186/1749-8104-5-29

**Published:** 2010-11-02

**Authors:** Jung-Hwan Choi, Mei-Yee Law, Chi-Bin Chien, Brian A Link, Rachel OL Wong

**Affiliations:** 1 Department of Biological Structure, University of Washington, Seattle, WA 98195, USA; 2 Department of Neurobiology and Anatomy, Neuroscience Program, Brain Institute, University of Utah, Salt Lake City, Utah 84132, USA; 3 Department of Cell Biology, Neurobiology and Anatomy, Medical College of Wisconsin, Milwaukee, WI 53226, USA

## Abstract

**Background:**

Many neurons in the central nervous system, including retinal ganglion cells (RGCs), possess asymmetric dendritic arbors oriented toward their presynaptic partners. How such dendritic arbors become biased during development *in vivo *is not well understood. Dendritic arbors may become oriented by directed outgrowth or by reorganization of an initially unbiased arbor. To distinguish between these possibilities, we imaged the dynamic behavior of zebrafish RGC dendrites during development *in vivo*. We then addressed how cell positioning within the retina, altered in *heart-and-soul *(*has*) mutants, affects RGC dendritic orientation.

**Results:**

*In vivo *multiphoton time-lapse analysis revealed that RGC dendrites initially exhibit exploratory behavior in multiple directions but progressively become apically oriented. The lifetimes of basal and apical dendrites were generally comparable before and during the period when arbors became biased. However, with maturation, the addition and extension rates of basal dendrites were slower than those of the apical dendrites. Oriented dendritic arbors were also found in misplaced RGCs of the *has *retina but there was no preferred orientation amongst the population. However, *has *RGCs always projected dendrites toward nearby neuropil where amacrine and bipolar cell neurites also terminated. Chimera analysis showed that the abnormal dendritic organization of RGCs in the mutant was non-cell autonomous.

**Conclusions:**

Our observations show that RGC dendritic arbors acquire an apical orientation by selective and gradual restriction of dendrite addition to the apical side of the cell body, rather than by preferential dendrite stabilization or elimination. A biased arbor emerges at a stage when many of the dendritic processes still appear exploratory. The generation of an oriented RGC dendritic arbor is likely to be determined by cell-extrinsic cues. Such cues are unlikely to be localized to the basal lamina of the inner retina, but rather may be provided by cells presynaptic to the RGCs.

## Background

Understanding how dendritic arbors of neurons are shaped during circuit assembly *in vivo *remains a key goal in developmental neurobiology [1]. Many neurons in the central nervous system, including Purkinje cells [2], retinal ganglion cells (RGCs) [3,4], layer IV neurons of the somatosensory cortex [5,6], mitral cells in the rodent olfactory bulb [7-10] and projection neurons in the fly olfactory system [11,12], form asymmetric dendritic arbors that are directed toward their presynaptic partners. Such asymmetric shapes of dendritic trees facilitate investigations into the cellular mechanisms that regulate the patterning and connectivity of the dendritic arbor.

Asymmetric or highly oriented dendritic arbors could be attained by two distinct mechanisms. Neurons may target their dendrites toward their presynaptic partners from the earliest stages of dendritic elaboration. This appears to occur in neurons of the chick nucleus laminaris [13,14], tectal neurons [15,16], and projection neurons in the fly olfactory system [17]. Recent studies have provided a wealth of information concerning the molecular and cellular mechanisms that underlie such dendritic 'targeting' [15-17]. Alternatively, neurons may initially project their dendrites in random directions and subsequently undergo remodeling to acquire a highly oriented arbor. Classic examples of neurons adopting this strategy are Purkinje cells [2,18] and spiny stellate cells of the barrel cortex [5,6].

For cells that undergo dendritic reorganization, it has not been possible in past studies to ascertain the dynamic rearrangement/remodeling events that would lead to the formation of a biased arbor. This is largely because major classes of neurons comprise many subtypes [19,20] that are not easily distinguished at earlier stages of development. Thus, it is difficult to discern whether differences in dendritic morphology between neurons at distinct ages reflect the maturation of the dendritic arbor, or variations in morphology amongst different subtypes. In order to determine whether an oriented dendritic arbor is acquired by selective addition, elimination, or stabilization of dendrites, time-lapse imaging of dendrites from their initial outgrowth until the arbor is oriented is necessary.

**Figure 1:**
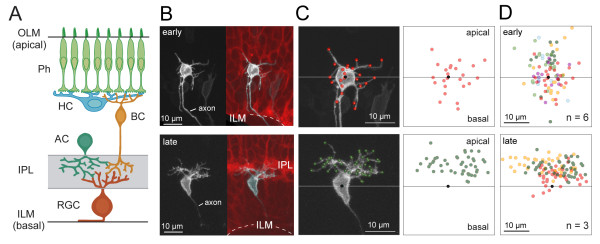
**Mature retinal ganglion cells project dendritic arbors apically toward presynaptic partners**. **(A) **Schematic showing retinal ganglion cells (RGCs) in the adult vertebrate retina orient their arbors toward their presynaptic partners, amacrine cells (ACs) and bipolar cells (BCs). Their synaptic contacts are confined to the inner plexiform layer (IPL). RGC axons exit basally from the cell bodies before traversing along the inner limiting membrane (ILM) and exiting the eye. Ph, photoreceptors; HC, horizontal cell; OLM, outer limiting membrane. **(B) **RGCs were imaged from less differentiated regions of the retina ('early') and compared to those from more differentiated regions ('late'). Less differentiated regions were distinguished from more differentiated regions by the absence or presence, respectively, of an IPL visualized in the background of *Q01 *transgenic animals (red; maximum projection of three optical planes centered at the RGC soma). RGCs were labeled by transient expression of *isl2b:MGFP *(greyscale). Dotted line, ILM. The images in the top and bottom panels were acquired at 51 hours post-fertilization (hpf) and 60 hpf, respectively. **(C) **Dendritic tips, neurites > 1 μm, for the examples in (B) are marked by colored dots. The horizontal line runs parallel to the ILM and crosses the approximate center of the cell body (black dot). **(D) **Summary of the spatial distributions of dendritic tips for the measured population (n = number of cells). Each color represents a different cell.

RGCs in the adult vertebrate retina orient their dendritic arbors toward their presynaptic partners, amacrine cells and bipolar cells, and form synaptic connections in the inner plexiform layer (IPL) (Figure 1A). However, some RGCs in initial stages of dendritogenesis have been reported to project dendrites in random directions, although an apically oriented arbor emerges with maturation [3,21-26]. We thus used RGCs as a model system to visualize how an oriented dendritic arbor arises from a multipolar arrangement. Because it is possible to track individual RGCs in the rapidly developing zebrafish retina over hours to days [27], we are able to visualize dendritic growth and remodeling across the entire period of development for these cells *in vivo*. Many RGCs could be found with oriented dendritic arbors by 2.5 days post-fertilization (dpf) [27]. We therefore imaged zebrafish RGCs *in vivo *between 40 and 60 hours post-fertilization (hpf) in order to determine how these cells dynamically progress from initial stages of dendritic outgrowth to acquire an apically oriented dendritic arbor. We then analyzed the dynamic behavior of dendrites to discern whether selective elaboration and/or stabilization of apical dendrites lead to an apically directed dendritic arbor.

Cues from the extracellular environment have been shown to influence the orientation of dendritic arbors *in vitro *and *in vivo *[28,29]. Whether the extracellular environment dictates the apical direction of the RGC dendritic arbor *in vivo *is, however, unclear. For example, the basal lamina has been shown to influence the direction of axon emergence in RGCs [30-34], but whether this lamina is eventually repulsive to dendrites is not known. In preparations in which RGCs are seeded onto frozen slices of chick retina, Müller glial endfeet inhibit dendritic outgrowth whereas glial somata allow or promote dendritic outgrowth [31,34]. However, it has not been determined whether Müller glia influence dendritic patterning of RGCs *in vivo*. Another possibility is that an attractive cue is presented by neurons that are presynaptic to RGCs, such as amacrine cells [35], which elaborate their neurites towards RGCs before RGC dendritic arbors become apically oriented [36].

Experimental conditions in which RGCs are mislocalized within the retina could thus provide further insight into the mechanisms controlling dendritic patterning of these neurons. We thus examined the organization of RGC dendritic arbors in the *heart-and-soul *(*has*) mutant in which a mutation in the aPKCλ gene results in a carboxy-terminal truncated protein product lacking kinase activity [37]. In this mutant, cells of the major retinal classes are often positioned in a local cellular environment that differs from their native environment. Furthermore, although *aPKCλ *mRNA is initially expressed throughout the retina in neuroepithelial cells, protein expression is confined to the apical surface at 32 hpf and not present in RGCs when they differentiate dendrites [38]. Thus, if the local environment regulates dendritic orientation in RGCs, we expect to find that mislocalized RGCs in the *has *mutant misorient their dendritic arbors or fail to develop an oriented arbor altogether.

## Results

### RGC dendrites initially project both apically and basally

Cells in different regions of the retina vary in their maturity due to a developmental wave that progresses from the central to peripheral retina [39-41]. We visualized the morphology of individual RGCs using mosaic expression of membrane-localized fluorescent proteins driven by RGC-specific promoters. By examining RGC dendritic morphology at various stages, we found that most RGCs initiate dendritic development between 36 and 60 hpf, although cell division and differentiation were still occurring at 60 hpf at the front of the developmental wave in peripheral retina. To determine how dendritic orientation is established with cellular maturation, we compared RGCs in relatively immature regions to those from more mature regions within or across animals. All labeled cells clearly bore axons, confirming their identity as RGCs. Neurites other than axons that were seen at all stages examined will be referred to as 'dendrites'.

**Figure 2:**
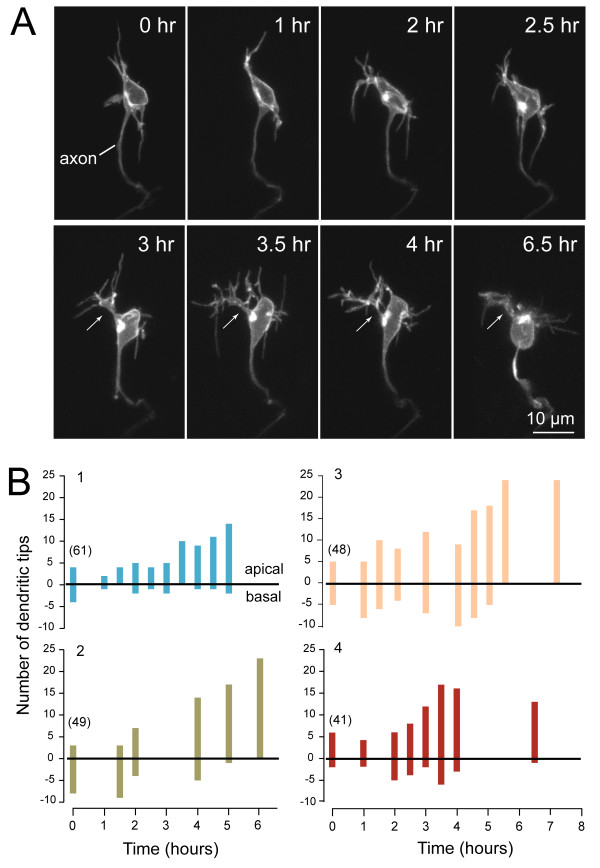
***In vivo *time-lapse imaging showing transition of a retinal ganglion cell dendritic arbor from unoriented to apically oriented**. **(A) **Maximum intensity projections of multiphoton image stacks of an RGC at successive time-points, beginning at 40 hpf. The arrows indicate a primary dendrite. Cell is oriented with apical direction upwards. See Additional file 1 (movie of the time-lapse). **(B) **Quantification of the dendritic behavior of four representative RGCs over several hours, showing progressive orientation of the dendrites towards the apical direction. Numbers at the first time-point indicate the age (hpf) of the embryo when the time-lapse commenced. Because the retina shows a centroperipheral gradient in maturation, cells at various stages of maturation can be found within an eye.

Here, the relative 'maturity' of a retinal region was ascribed by the presence (more mature) or absence (immature) of an IPL based on cell membrane staining (Figure 1B). In contrast to more mature regions of the retina, RGCs in immature regions of the retina possessed many basal dendrites as well as apical dendrites (Figure 1B-D). Basally directed dendrites emerged either directly from the cell body or from lateral processes. To visualize and monitor how dendrites become apically biased in their projection towards the IPL with maturation, time-lapse imaging (30-minute to 2-hour intervals) of individual RGCs was conducted. At the earliest time-points, most dendrites emerged from multiple sites on the cell body and did not branch extensively (Figure 2A; Additional files 1 and 2). The arbors became more apically biased with development as the number of basal dendrites progressively declined (Figure 2A, B; Additional file 1). The transition from an unbiased to a biased arbor typically occurred within 4 to 12 hours (7.3 ± 0.8 hours; n = 10 cells; examples provided in Figure 2B). Our time-lapse recordings also demonstrated that primary dendrites were apparent before the arbors became completely apically biased (examples are indicated by arrows in Figure 2; n = 7 cells). These primary processes often appeared to emerge from sites on the cell body that had earlier showed relatively profuse dendritic growth.

### Dynamic behavior of apical and basal dendrites

**Figure 3:**
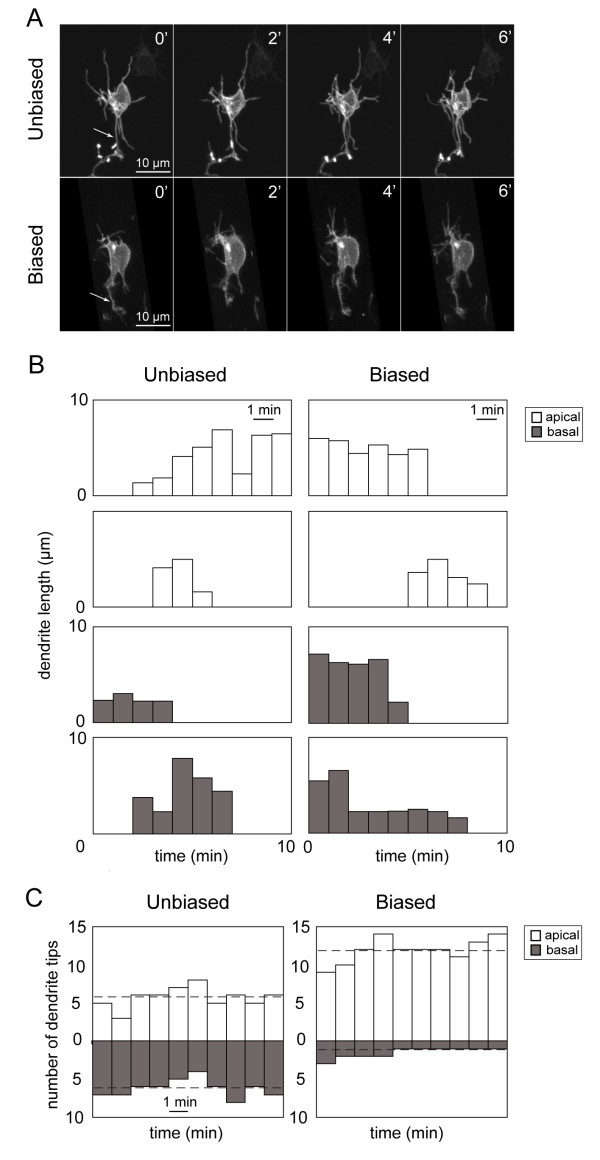
**Dynamic behavior of apical and basal dendrites of developing retinal ganglion cells**. **(A) **Example of a time-lapse recording (time elapsed in minutes) from an 'unbiased' and a 'biased' RGC showing extension and retraction of processes. Arrows indicate axons. **(B) **Examples of the dynamic behavior of individual apical dendrites (white) and basal dendrites (grey) over time showing dendritic length over time and dendritic lifetimes. Motility rates (Figure 4) of individual dendrites were calculated from such plots. **(C) **Examples showing how the numbers of apical and basal tips change over time (10 minutes of recording). Shown here are the plots for a cell with an unbiased arbor and a cell with a biased arbor. The dotted line indicates the average number of apical or basal dendrites of the cell during the recording period.

If RGCs initially extend both basal and apical dendrites, how do dendrites become increasingly apically biased during maturation? Possible explanations include several potential differences in the dynamic behavior of dendrites. Apical dendrites may be added more quickly than basal dendrites (greater addition rate). Apically projecting dendrites may be more stable than basal dendrites (greater lifetime) such that, at any time-point, the cell has more apical rather than basal dendrites. Apical dendrites may extend more rapidly and/or retract more slowly than basal dendrites (differing extension and retraction rates). To determine what differences in dendritic dynamics occur between apical and basal dendrites, we performed more rapid time-lapse recordings, acquiring images at 1- or 2-minute intervals (Figure 3A). Rates of dendrite addition, lifetime, and motility rates were quantified for apical and basal dendrites for RGCs with unbiased arbors (defined as cells in which the number of apical dendritic tips is between 40 and 60% of the total number of tips; see Additional file 3 for an example) and for RGCs with biased arbors (those with more than 60% apical dendrite tips; Additional file 4). Characterization of behaviors for individual dendrites is shown in Figure 3A, B.

For RGCs with unbiased arbors, we found that at any one time-point, not only can apical dendrites outnumber basal dendrites of the same cell, but basal dendrites can outnumber apical dendrites (see Figure 3C for an example). However, on average, the numbers of apical and basal dendritic tips were not significantly different (n = 7 cells; *P *= 0.11; Wilcoxon signed rank test) over the recording period. For RGCs with biased arbors, the number of apical dendrite tips always exceeded the number of basal tips at each time-point (see Figure 3C), and on average, the numbers of apical and basal tips were significantly different (n = 8 cells; *P *= 0.008; Wilcoxon signed rank test).

**Figure 4:**
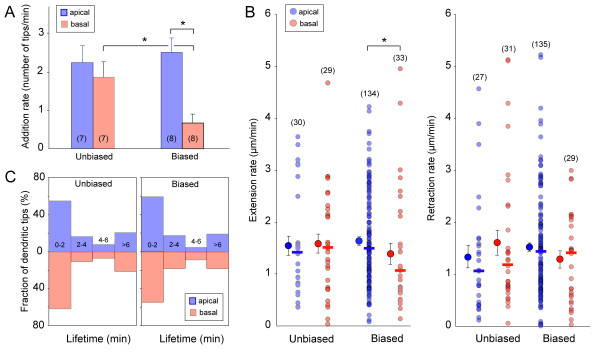
**Apical and basal dendrites differ in their rate of addition as a biased arbor emerges**. **(A) **The rate of dendritic tip addition (see Materials and methods) was significantly different between apical and basal dendrites in cells with biased arbors but not in cells with unbiased arbors. Numbers on the bar graphs indicate the number of cells (n) analyzed. Error bars represent standard error of the mean (SEM). *P < 0.05. **(B) **Extension rates of apical and basal dendrites were significantly different in cells with biased arbors (*P *< 0.05), but not in cells with unbiased arbors. Retraction rates were not significantly different (*P *> 0.05 for all plots) between apical and basal dendrites of cells within a stage (biased or unbiased). Numbers in parentheses indicate the number of dendrites (n) measured. Solid symbols are median values of the respective distributions of the raw values plotted on the adjacent columns. Error bars represent SEM. **(C) **Lifetime distributions of apical versus basal dendrites did not differ for cells with unbiased or with biased arbors.

We next carried out quantitative comparisons of various measures of the dynamic behaviors of RGC dendrites at the two developmental stages (unbiased and biased). First, comparison of dendrite addition rates showed that cells with unbiased arbors did not exhibit a significant difference in addition rates between apical and basal dendrites (Figure 4A; n = 7 cells; *P *= 0.456; Mann-Whitney U test). In contrast, cells beginning to demonstrate a biased arbor showed a significant difference between apical and basal tip addition rate (Figure 4A; n = 8 cells; *P *= 0.003). However, the average addition rate of apical dendritic tips was not significantly different between RGCs with unbiased arbors and RGCs with biased arbors (*P *= 0.78). Apical tips therefore appear to be added at a relatively constant rate across the RGC developmental stages we examined. In contrast, there was a statistical difference between the rate of basal tip addition in cells across the two stages (*P *= 0.04). We observed that at a later stage when RGCs possessed only apical dendrites, they rarely exhibited transient basal dendrite outgrowth. This implies that the rate of basal dendrite addition gradually declines with maturation. Thus, a developmental decrease in basal dendrite addition, rather than an increase in apical dendrite addition, likely contributes to the formation of a biased dendritic arbor.

We then determined whether differences in addition rates could arise from differential motility rates of apical and basal dendrites. For example, a relatively greater retraction rate of basal dendrites compared to apical dendrites, or a slower extension rate, could conceivably lead to an apically biased arbor. We found that dendrite extension rates did not significantly differ between apical and basal dendrites of cells with unbiased arbors (Figure 4B; *P *= 0.97; 3 cells). In contrast, extension rates were significantly different between apical and basal dendrites of cells developing a biased arbor (Figure 4B; *P *= 0.03; 5 cells). There was no significant change in apical or basal dendrite extension rates when comparing between unbiased and biased arbors (apical, *P *= 0.49; basal, *P *= 0.30). There was also no significant difference between apical and basal dendrite retraction rates for either unbiased or biased arbors (Figure 4B; unbiased, *P *= 0.45, 3 cells; biased, *P *= 0.26, 5 cells) nor was there a difference in retraction rates between unbiased and biased arbors (apical, *P *= 0.13; basal, *P *= 0.56).

The appearance of a biased arbor may also result from apical dendrites having longer lifetimes than basal dendrites. Therefore, to compare the relative lifetimes of apical and basal dendrites of RGCs at the two stages of dendritic development, we measured lifetimes of dendrites that appeared and disappeared within the recording period. The majority of our recordings ranged from a total imaging period of 10 to 20 minutes (images acquired every 1 to 2 minutes). Figure 4C plots the distribution of dendritic tips whose lifetimes could be defined (1 to 6 minutes), and those that were present for at least 6 minutes (> 6 minutes). Dendrites that were present for at least 6 minutes within the recording period but whose appearance or disappearance was not visualized were also assigned a lifetime of > 6 minutes. We found that, for cells with an unbiased arbor, 80% of all apical dendritic tips and 79% of all basal dendritic tips measured (176 apical, 157 basal dendritic tips from 8 cells) had lifetimes between 1 and 6 minutes (Figure 4C). For cells with a biased arbor, 81% of all apical tips and 82% of all basal tips measured (148 apical, 44 basal dendritic tips from 5 cells) had lifetimes between 1 and 6 minutes. Thus, the majority of apical and basal dendrites were relatively transient at both stages, suggesting that an apically biased dendritic arbor emerges even before dendrites have started to become stabilized.

### Mislocalized retinal ganglion cells have abnormally directed dendrites

**Figure 5:**
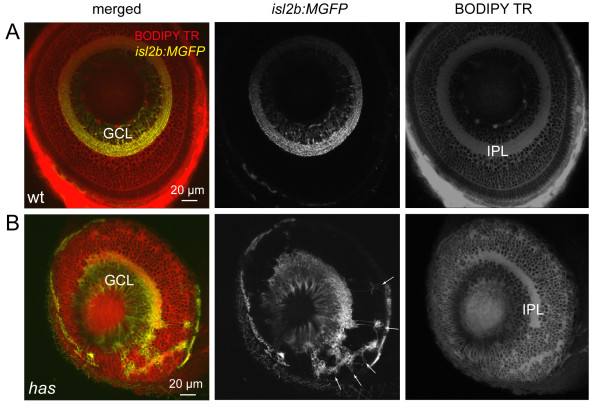
**Some retinal ganglion cells are mislocalized in the *has *mutant**. **(A) **Normal localization of RGCs to the ganglion cell layer (GCL) in the *isl2b:MGFP *line. BODIPY Texas Red (TR) labeling clearly reveals the cell body-free inner plexiform layer (IPL). Wt, wild type. **(B) **Distribution of RGCs in the *isl2b:MGFP; has *mutant retina. Arrows point to mislocalized RGCs.

**Figure 6:**
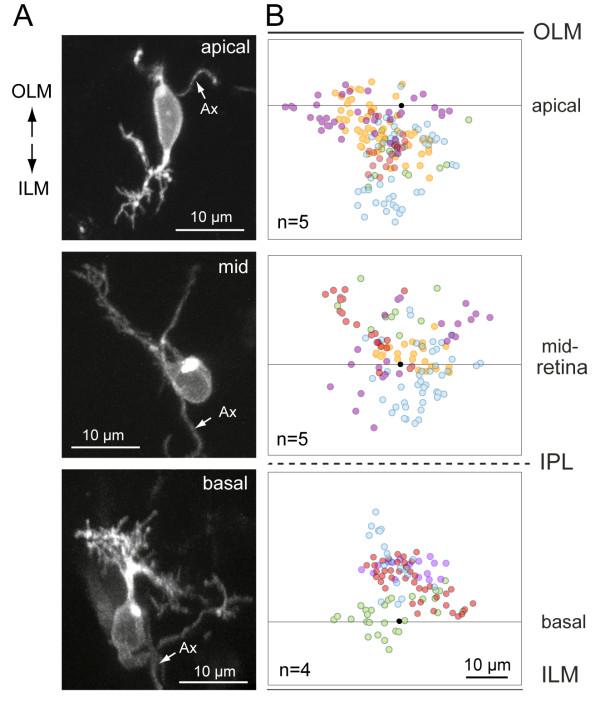
**Distribution of dendritic tips in mislocalized retinal ganglion cells in the *has *retina**. RGCs were labeled either by transient expression of *isl2b:MXFP *in *has *animals or visualized in *brn3c:MGFP; has *animals. Ax, axon. **(A) **Examples of isolated RGCs at various depths of the *has *retina. The cell was oriented in three dimensions as described in the Materials and methods. **(B) **Summary of the distributions of dendritic tips for several RGCs at the various depths. Black dots represent the center of the soma; the line through the dot is approximately parallel to the ILM or OLM. Each color represents a separate RGC. Red dots are tips from the cells shown in the left column.

To determine whether RGCs can develop an oriented dendritic arbor independent of their cell body position within the retina, we compared the dendritic arbors of RGCs in wild-type animals to those in *has/aPKCλ *mutants (Figure 5A, B). In mutants, the somata of RGCs were found not only at the usual location in the ganglion cell layer (GCL) adjacent to the inner limiting membrane (ILM), but also near the apical surface (outer limiting membrane (OLM)) and in the middle of the retina, consistent with previous studies [30,38]. In some cases, mislocalized RGC arbors could be visualized in isolation (Figures 5B and 6). For such cells, we observed a long process heading away from the cell body that we presumed to be the axon (Additional file 5); a few of these processes bore a short collateral. In contrast, neurites that branched frequently close to the cell body and often formed arbors were presumed to be dendrites (Additional files 5 and 6). We found that basally located RGCs near the ILM had dendritic arbors that were oriented apically as in wild-type animals. In contrast, apically located RGCs generally projected their dendrites basally, away from the OLM. RGCs located near the middle of the retina often possessed biased or oriented dendritic arbors but such arbors did not project in a consistent direction across cells. A few RGCs localized to the middle of the retina projected dendrites in multiple directions, not forming a clearly biased arbor. A summary of the distribution of dendritic tips for individual RGCs at various depths in the *has *retina is provided in Figure 6.

**Figure 7:**
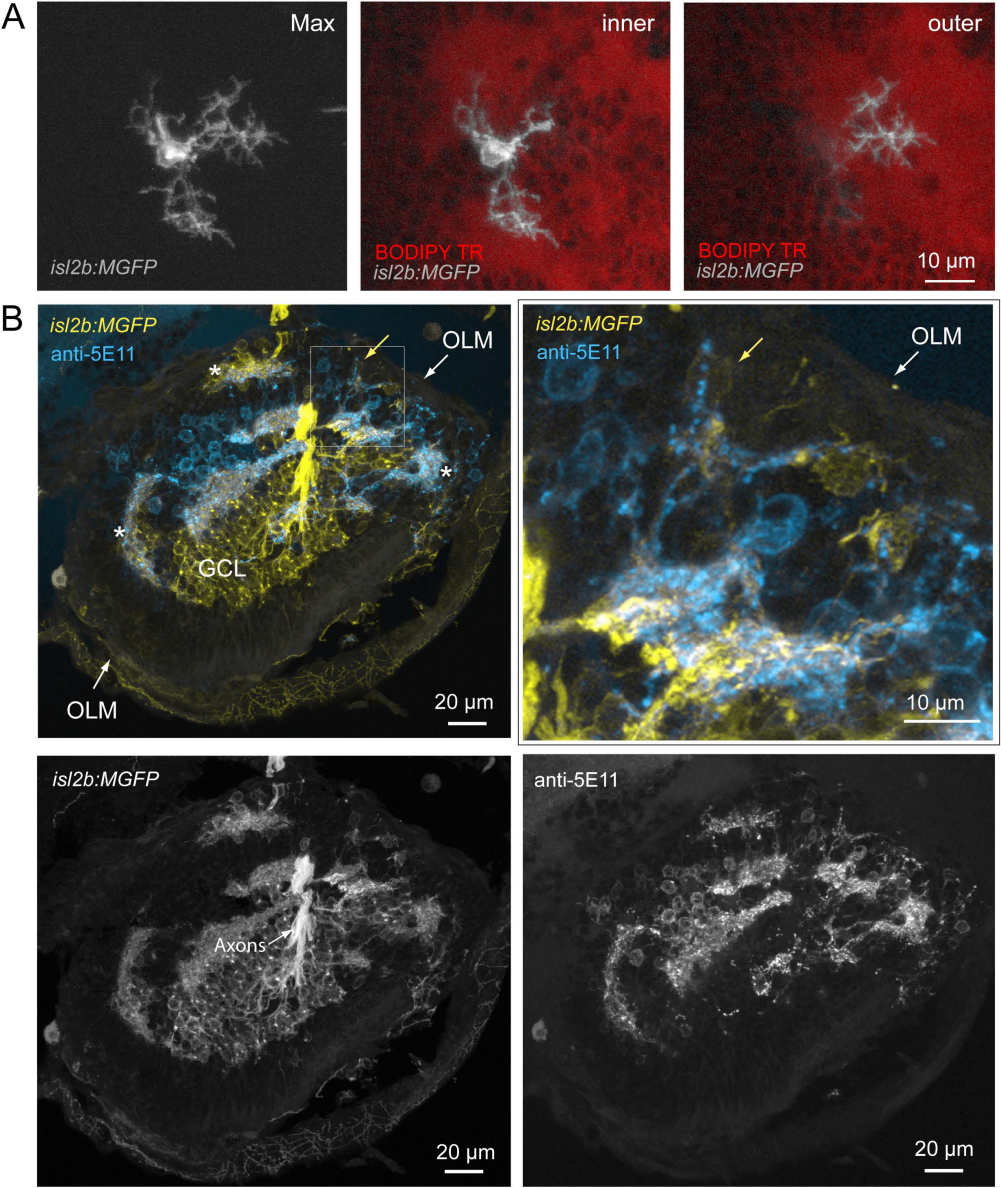
**Misplaced retinal ganglion cells project dendrites towards neuropil comprising amacrine cell neurites**. **(A) **Example of a mispositioned RGC with dendritic terminals projecting to cell body-free regions, putative neuropil, at different retinal depths (Max, maximum projection of image stack). While two arbors (inner) terminate close to the cell body (towards the ILM) within a neuropil region, one arbor projects into a deeper (towards the OLM) part of this uneven neuropil, towards the back of the eye (outer). See Additional file 6 for viewing sequential image planes of the three-dimensional reconstruction of the cell. **(B) **Colocalization of RGC dendrites and amacrine cell neurites also occur in ectopic locations (asterisks). RGCs are labeled by expression of GFP in the *isl2b:MGFP *line crossed into the *has *background. Amacrine cells are immunolabeled by anti-5E11 in this frozen section. Higher magnification of the boxed area shows processes of a ganglion cell (yellow arrow) colocalizing with 5E11 staining (cyan). OLM, outer limiting membrane; GCL, ganglion cell layer.

**Figure 8:**
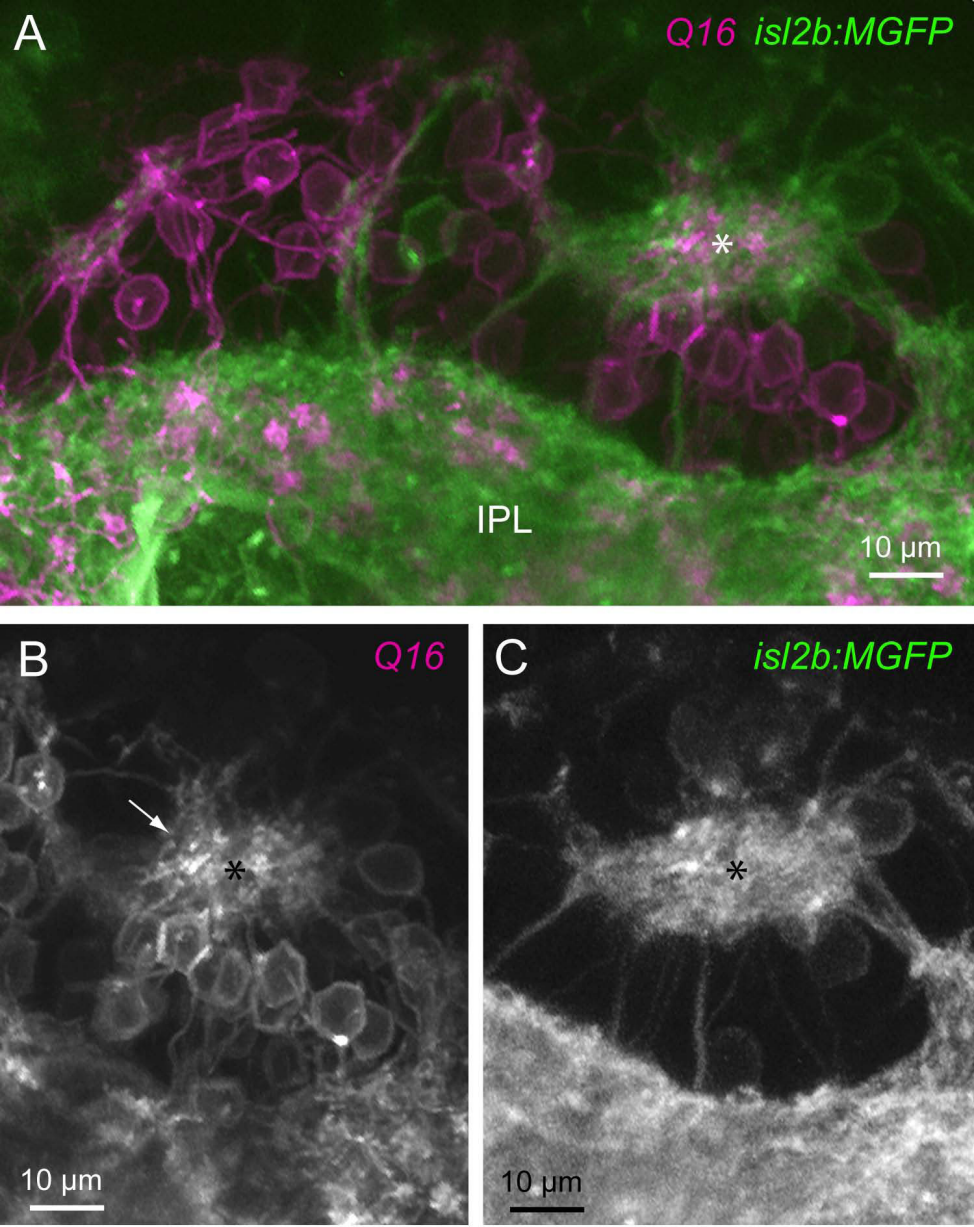
**Ectopic neuropil in *has *mutants comprise processes of retinal ganglion cells and bipolar cells**. **(A) **Multiphoton reconstruction of the processes of RGCs (*isl2b:MGFP*) and bipolar cells (*Q16*). An ectopic neuropil is marked by the asterisk. A continuous band of RGC and bipolar cell processes closest to the ILM is denoted here as the inner plexiform layer (IPL). **(B) **Bipolar cell processes (arrow) projecting to the ectopic neuropil (asterisk) are apparent. **(C) **The ectopic neuropil also contains processes of RGCs.

The dendrites of mislocalized RGCs oriented towards regions devoid of cell bodies, which appear to form ectopic neuropil, as visualized by BODIPY Texas Red labeling of cell membranes (Figure 5B). In some cases, dendritic terminals could be found to project towards neuropil-like regions at different retinal depths (for example, Figure 7A; Additional files 5 and 6). We asked whether these cell body-free regions comprise neurites of cells that normally contact RGC dendrites, especially amacrine cells, which extend neurites concurrently with dendrite outgrowth in RGCs [36]. Immunostaining for amacrine cells in the *has *retina with XFP-labeled RGCs (see Materials and methods) clearly demonstrates the presence of amacrine neurites in patches to which RGC dendrites project (Figure 7B; nine of nine neuropil-like regions, two retinas). We also asked whether processes of bipolar cells contribute to the ectopic neuropil containing neurites of RGCs. Simultaneous visualization of bipolar cells [42] and RGCs in the *Q16/isl2b:MGFP *double transgenic line suggests that bipolar cell processes do colocalize with RGC neurites in ectopic locations (Figure 8). Thus, it appears that RGC dendrites orient towards locations where at least some amacrine cells and bipolar cells contribute neurites.

**Figure 9:**
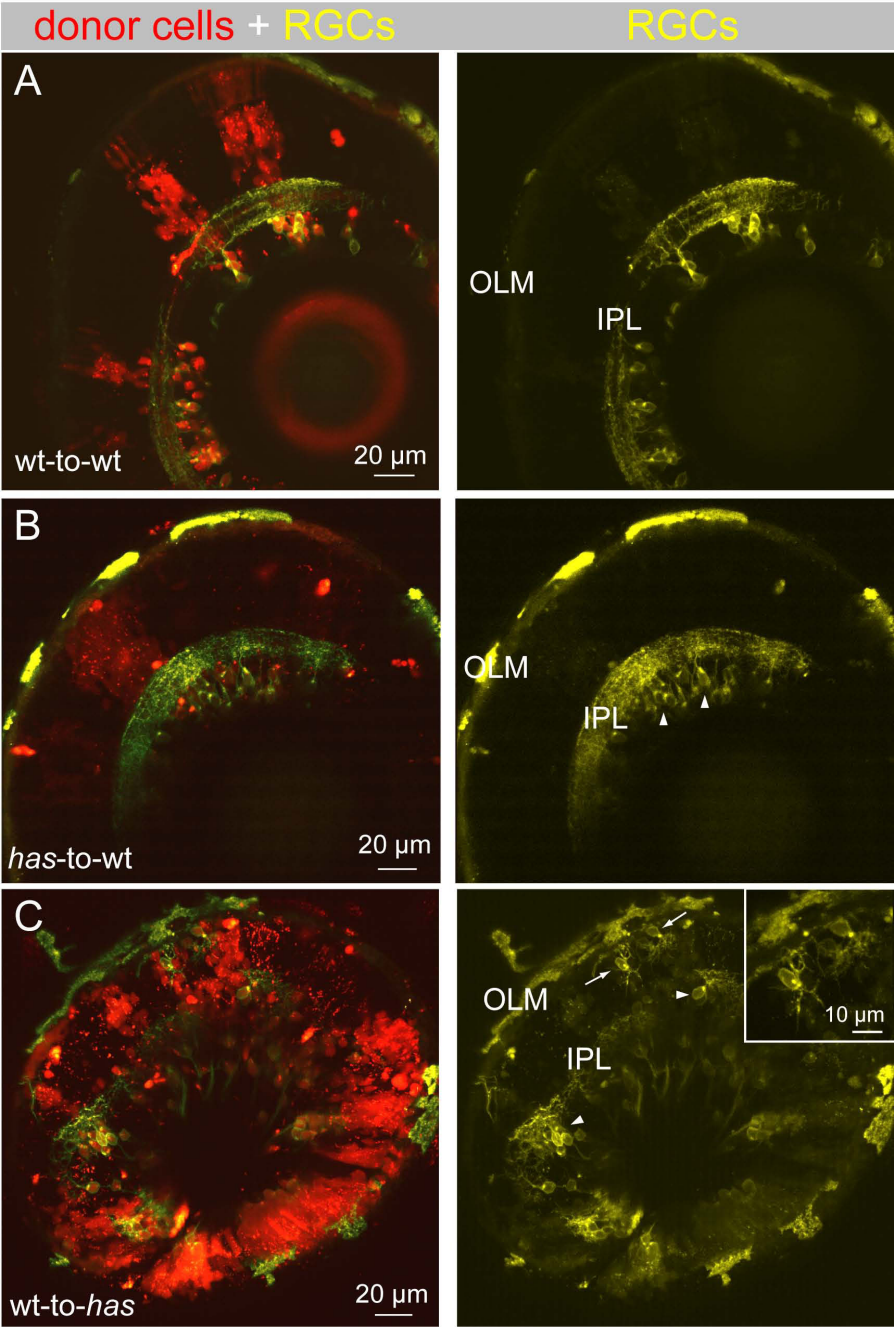
**Dendritic misprojections of ectopically located retinal ganglion cells in the *has *mutant is non-cell autonomous**. In each chimera, donor cells were labeled by a lineage tracer (red), fluororuby. Donor cells were always taken from the *isl2b:MGFP *line (yellow), either in a wild-type (wt) or *has *background, to better visualize dendrites. **(A) **Wild-type donor cells transplanted to a wild-type host. RGC dendritic arbors were oriented toward the OLM and stratified within an IPL. **(B) ***has *donor cells transplanted to a wild-type host. All *has *RGCs were located in the GCL (arrowheads). **C**. Wild-type donor cells transplanted to a *has *host. Most RGCs were located in the GCL (arrowheads), but a few cells were located ectopically (arrows). Inset shows higher magnification of the misplaced RGCs indicated by the arrows.

Finally, we considered the possibility that the misoriented arbors of RGCs in *has *were caused by a cell autonomous effect of the mutation because aPKCλ is involved in defining cell-polarity in many cell types [43-47]. To ascertain whether this is the case, we performed mosaic analysis of *has *animals (Figure 9). *has *mutant cells were transplanted to wild-type (wt) hosts to create a *has*-to-wt chimera. In these chimeras, donor RGCs all localized properly to the GCL (Figure 9B) and all RGCs extended their dendritic arbors apically toward the IPL (three animals). Their arbors were laminated within the IPL, similar to wt-to-wt transplants (Figure 9A; six animals). In contrast, when wild-type cells were transplanted into *has *hosts (wt-to-*has*), donor RGCs exhibit phenotypes similar to non-chimeric *has *mutants (six animals). Donor RGCs were located next to the apical surface, in the middle of the retina, as well as near the basal surface (Figure 9C; n = 9 mislocalized cells in isolation imaged). Taken together, these results suggest that aPKCλ plays a non-cell autonomous role in RGC dendritic orientation.

## Discussion

### Early outgrowth and patterning of retinal ganglion cell dendrites

Previous studies that have examined the early development of RGCs have provided a detailed view of axogenesis in these neurons across species [4,21-24,26,30,48-52]. In some of these studies, dendritogenesis was also observed [21-26,30,49-52]. Like RGCs in other species, we found that at a stage after axonogenesis, zebrafish RGCs project neurites both apically and basally prior to establishing an apically biased dendritic arbor. A developmental progression from a multipolar to a highly polarized dendritic arbor also occurs in other central nervous system neurons, such as Purkinje cells in the rat cerebellum [2,18] and layer IV stellate neurons of the mouse barrel cortex [5]. However, previous studies did not analyze what changes in dendritic dynamics could lead to the reshaping of dendritic projection patterns during maturation.

Here, comparison of the dynamic behavior of the apical and basal neurites of the zebrafish RGCs revealed that a relative decline in the addition rate of basal, compared to apical, dendritic tips coincided with the emergence of a biased dendritic arbor. Although it is not unexpected that addition of basal neurites should decrease with maturation of the RGCs, it was surprising that the addition rate in the apical direction did not increase during development. The emergence of a biased arbor may also be partly attributed to a relatively faster extension rate of apical dendrites compared to basal dendrites as arbors became oriented. Across the ages examined, the majority of apically projecting neurites were not stable, and in fact possessed lifetimes similar to those of the basal processes. Although many of the early apical neurites were transient, it appeared that once well-defined primary dendrites were established, neurite addition became concentrated at these processes.

Are there changes in the spatial distribution of cellular components that could account for the progressive biased outgrowth in the apical direction? One possibility is localization of the Golgi apparatus to the apical part of the RGC soma to trigger apically focused dendritic outgrowth. Electron microscopy studies carried out at different stages of RGC differentiation suggested that the Golgi localizes within the apical process or at the apical side of the cell body as RGCs migrate towards the GCL after their birth [3,48]. However, after migration to the GCL at the stage when an axon has extended but dendrites have not, the Golgi apparatus can be found at either the apical or basal side of the cell body. Time-lapse analysis in the future could provide further insight into the relationship between dendritic outgrowth and positioning of the Golgi. Thus, unlike hippocampal neurons in culture [53], the Golgi in RGCs may play a supportive, rather than an active, role in patterning dendritic orientation.

Another factor that could regulate the orientation of the RGC dendritic arbor is the axon initiation site. Since RGC axons grow out before dendrites [22,24,54], axon exit sites may establish the basal side of the cell body as a reference point [30] despite an initial phase of multipolar dendritic elaboration. *In vitro*, RGCs can form a dendritic arbor opposite their axons [30], suggesting that the relationship between axon and dendritic orientation is maintained in the absence of extrinsic cues. However, *in vivo*, RGC dendritic arbors are not always oriented opposite their axon exit sites [55-57]. In the wild-type zebrafish retina, some RGCs are initially located with their somata in the inner nuclear layer (data not shown). These displaced RGCs projected both their axon and dendritic arbor basally. In the *has *retina, there were RGCs close to the OLM (approximately 10%) with both their axon and dendrites projecting apically, as well as examples of mislocalized cells with axons and dendrites projecting basally (see Additional file 5 (cell to the right) for an example). Together, these observations suggest that the initial axon trajectory and dendritic orientation of RGCs are likely to be independently controlled, most likely by extrinsic cues, similar to the axons and apical dendrites of cortical pyramidal neurons [28].

### Influence of cell-positioning on retinal ganglion cell dendritic orientation

Dissociated zebrafish RGCs are capable of forming a polarized dendritic arbor, suggesting that these neurons have an intrinsic capability to form asymmetric dendritic arbors [30]. We found that in the *has *retina, most mislocalized RGCs display a biased dendritic arbor. The orientation of dendritic arbors of these ectopically located cells was, however, not consistent relative to the apical or basal surfaces of the retina, including some tangential orientations. Thus, it appears that the stereotypical apical orientation of RGC dendritic arbors in the wild-type retina is unlikely to be determined by the proximity of the cells to the basal lamina, which has previously been shown to influence RGC axon trajectories [30]. Nevertheless, environmental cues, rather than intrinsic cues, are still likely to be at play because all mislocalized RGCs in the *has *mutant projected dendrites terminating in cell-free zones resembling neuropil.

What environmental cues may then orient RGC dendrites? One possibility is that presynaptic partners may promote dendritic outgrowth towards the forming IPL. In addition to demonstrated roles for diffusible factors [28], a role for contact with presynaptic cells can be found in several systems. For example, when the number of granule cells that project parallel fibers to innervate the dendrites of Purkinje cells is reduced greatly in *weaver *mutant mice or by X-irradiation, Purkinje cells orient their arbors not only apically but in lateral or basal directions [58-61]. Also, when afferents onto ventral dendrites of nucleus laminaris neurons of the chick auditory brainstem are cut, dorsal dendrites extend whereas ventral dendrites retract [62], in this case creating an abnormally asymmetric arbor.

During RGC dendritic outgrowth, the first presynaptic cells present are amacrine cells. Amacrine cell bodies are localized opposite to the GCL by at least 39 hpf [36]. The arrival of amacrine cells and extension of their neurites towards the RGCs coincides with the period in which RGC dendritic arbors transition from being unbiased to biased. Furthermore, amacrine cells, which express *sonic hedgehog *(*shh*) mRNA in wild-type retina, are sufficient to restore local lamination in the *shh *mutant retina [63]. Previous observations from dissociated rat retinal cell cultures have suggested that amacrine cell membranes promote dendrite outgrowth in RGCs [35]. Thus, amacrine cells may provide cues that promote apically directed extension of RGC dendrites over time. In the *has *mutant, the total number of amacrine cells appears unchanged [38]. We observed that mispositioned RGCs project dendrites towards neuropil-like regions that contain amacrine cell neurites as well as bipolar cell processes. Certainly, orientation of amacrine cell neurites and bipolar cell axons towards RGCs does not require the presence of RGCs themselves [64]. Establishing the importance of amacrine cells in helping direct dendritic bias in RGCs would require analysis of RGC dendrites in mutants that lack amacrine interneurons - for example, *foggy *[65], in which GABAergic and dopaminergic amacrine cells fail to differentiate but a properly located GCL persists. Although bipolar cells are thought to differentiate later than amacrine cells, their processes may also contribute to stabilizing RGC dendrites within the neuropil. While the factors that orient RGC dendrites towards their presynaptic partners have yet to be revealed, our current results support the possibility that this stereotypical arrangement of the RGC dendritic arbor is shaped by interactions with the local environment.

## Conclusions

Our *in vivo *imaging observations suggest that a reduction in the addition rate of basal dendrites primarily contributes to the establishment of an apically oriented arbor with maturation. Furthermore, the extracellular environment directs the patterning of RGC dendrites such that RGC dendrites always project towards neurites of presynaptic cells, regardless of their cell body locations within the depth of the retina. Thus, it is unlikely that the inner limiting membrane provides cues for directing RGC dendritic arbors to orient apically.

## Materials and methods

### *In vivo *cell labeling methods

To specifically label individual RGCs, zebrafish embryos at the one-cell stage were injected with *-17.6isl2b:gap43-XFP *(referred to as '*isl2b:MXFP*'), *brn3c:gap43-XFP *(referred to as '*brn3c:MXFP*') [66], or *ath5:gap-gfp *(construct provided by WA Harris) [30] DNA plasmids (typically, 5 to 25 ng/μl in the pipette; picospritzer used to inject plasmids set at pressure 2 to 10 psi, pulse duration 100 to 500 ms). *isl2b *constructs were generated from an ISceI-*isl2b-*attR1-attR2-GFP-SV40 polyA-ISceI destination vector (#482) by Gateway cloning; for membrane-targeted cyan fluorescent protein (MCFP) and membrane-targeted yellow fluorescent protein (MYFP) versions, a second polyA signal was included before the GFP. MXFP constructs (M, membrane-targeted by a palmitoylation sequence; X, yellow, cyan or green; FP, fluorescent protein) allow better visualization of fine dendritic processes. Because melanophores within the retina appear between 25 and 28 hpf [67] and hinder imaging, the embryos were incubated in solution containing a pigmentation inhibitor, 1-phenyl-2-thiourea (PTU; 200 μM) in 0.3× Danieau's solution. To prevent movement of the embryo or larva during imaging, the animals were anesthetized using tricaine (MS-222, Sigma-Aldrich, St. Louis, Missouri, USA); 750 μM) in Danieau's solution and mounted in 0.5 to 1.0% low-melting point agarose.

Retinal landmarks were provided by two methods of visualizing cell membranes. First, we used a transgenic line that labels all cell membranes, *Tg (Pax6-DF4:gap43-CFP)^Q01^*, or *Q01 *line [36]. Alternatively, embryos were incubated in vital dye CellTrace BODIPY Texas Red methyl ester (200 μM, Invitrogen, Carlsbad, California, USA) [68] for 1 hour before imaging commenced. These methods allow visualization of the different cellular layers, as well as the synaptic neuropil. When these methods could not be used, differential interference contrast images of the outlines of the ILM and OLM were obtained.

### Immunocytochemistry

Larvae were fixed at 4 to 5 dpf with 4% paraformaldehyde and 2% sucrose in 0.1 M PBS for 2 hours at room temperature, transferred through a series of sucrose solutions at 5%, 10%, and 15% for 30 minutes each at room temperature, then incubated overnight in 20% sucrose at 4°C. Two to five fish were embedded in Optimal Cutting Temperature cryomedium (OCT, Ted Pella, Inc., Redding, California, USA) in cryomolds (Tissue-Tek, Seattle, Washington, USA) on dry ice. Cryosections were cut at 20 μm thickness using a cryomicrotome (Leica CM 1850) and incubated in 5% normal goat serum in 0.1 M PBS for 1 hour. Cell-specific antibodies for amacrine cells (mouse anti-5E11, 1:50; gift from James Fadool) were diluted in 5% normal goat serum (in 0.1 M PBS) containing 0.5% Triton X-100 and applied to sections overnight at room temperature. After washes in 0.1 M PBS, sections were incubated for 1 hour at room temperature in secondary antibodies (Alexa Fluor 633 goat anti-mouse, 1:1,000; Invitrogen), washed in 0.1 M PBS and cover-slipped in Vectashield (Vector Laboratories, Burlingame, California, USA).

### *has *mutants and transgenic lines

For all mutant analysis, the *has *m567 allele was used [38]. *has *mutants were identified by their curved body and pericardial swelling. Because *has *mutants died at 4 to 6 dpf and their development was delayed compared to wild-type fish, we acquired confocal or multiphoton images of RGC dendritic arbors at 4 or 5 dpf to observe their mature dendritic orientation.

In order to visualize RGCs in the *has *retina, two RGC transgenic lines, *Tg(-17.6isl2b:gap43-GFP)^zc20^*, referred to here as the *isl2b:MGFP *line, and *Tg(brn3c:gap43-GFP)^s365t ^*[66], or *brn3c:MGFP *line, were bred into the *has *mutant background. The *isl2b:MGFP *line was generated by injecting one-cell AB* wild-type embryos with DNA and I-SceI meganuclease, then screening for germline integration after raising to adulthood (M-YL and C-BC, unpublished data). The *isl2b:MGFP *line appears to label all RGCs, whereas the *brn3c:MGFP *line labels approximately 50% of RGCs [66]. Bipolar cells are labeled by expression of YFP in the *Tg(nyx:MYFP)^Q16 ^line *[42], or *Q16 *line. All transgenic lines and *has *mutants were kept in the *roy *mutant background, which lack reflective iridophores [69], so as to facilitate *in vivo *imaging.

### Mosaic analysis

Genetic mosaic embryos were generated by blastomere transplantation [70]. Donor embryos were injected with a lineage tracer, fluororuby-dextran (5% in 0.1 M KCl; Invitrogen, Carlsbad, California, USA), at the one-cell to eight-cell stage. Donor embryos were derived from the *isl2b:MGFP *transgenic line in the wild-type or *has *background, so that all donor cells would be labeled by fluororuby and donor RGCs would express membrane-targeted GFP (to assist visualization of the dendritic arbor). Host and donor embryos were manually dechorionated at 3 hpf. Five to forty donor cells were transplanted to the animal pole, which later becomes the eye and forebrain [67,71], of host embryos at 3.5 to 6 hpf (high stage to germ ring stage). Donor embryos from *has *heterozygote parents were kept until 3 dpf in order to determine which donors were *has *homozygotes. Three different types of chimeras were generated: wt (donors)-to-*has *(hosts), *has*-to-wt, and wt-to-wt.

### Image acquisition

Images were obtained using either a FV500/1000 Olympus confocal microscope (440 nm, 488 nm, 568 nm) or a custom-designed multiphoton microscope with a Ti:Sapphire laser (Spectra Physics, Mai Tai BB, Mountain View, California, USA; 880 to 890 nm). Excitation power was minimized to prevent phototoxicity and bleaching for the live-cell imaging experiments. The arbors of RGCs were imaged between 40 and 60 hpf, using a 60× water objective (Olympus, NA 1.1). Imaging was performed at 28°C using a heated, temperature-controlled chamber. For time-lapse analysis, image stacks capturing the entire dendritic arbor were acquired every 30 minutes to 1 hour for a total duration of 4 to 12 hours. To track the behavior of individual processes, image stacks were obtained more frequently, at 1-minute intervals for a total duration of 10 minutes, or at 2-minute intervals for a total duration of 20 minutes. Kalman line averaging was performed during image acquisition to reduce noise. Separation of the YFP from GFP signal was attained using a CFP/YFP filter block on the multiphoton microscope: YFP signal, but not GFP signal, is absent in the CFP channel, whereas GFP and YFP signal are detected in the YFP channel.

### Image processing and analysis

Images were reconstructed and analyzed with the image analysis software Metamorph (Molecular Devices, Sunnyvale, California, USA), Amira (Visage Imaging, Andover, Massachusetts, USA), and Imaris (Bitplane, St. Paul, Minnesota, USA). All images were rotated so that the apical surface (OLM) faced upwards and the basal surface (ILM) faced downwards. Rotation of three-dimensional images relative to the apicobasal axis was guided by generating a plane parallel to the ILM (or IPL). Specifically, vectors perpendicular to the ILM surface near the RGC of interest were created using the SurfaceNormal function in Amira. Then, a plane that is orthogonal to these vectors that crosses the center of the ganglion cell body is chosen as the plane parallel to the ILM, which divides the apical and basal side of the cell (Figure 1B). Any neurite terminating on the apical side of this plane was considered an apical 'dendrite'. Similarly, a neurite terminating on the basal side was considered a basal 'dendrite' (except for the axon). For apically located RGCs in the *has *mutant, we rotated three-dimensional images relative to the apicobasal axis by generating a plane parallel to OLM, rather than the ILM.

### Dendrite orientation and dynamics

Three-dimensional images acquired at different time-points were aligned in Amira based on cell body location and the axon trajectory. Dendritic tips (process length > 1 μm) are marked by a dot to obtain a spatial map of these tips in order to visualize overall dendritic orientation for individual cells. The center of the cell body is defined by the center of mass of the soma, obtained using Amira. Dendrite length was estimated in the three-dimensional reconstructions by the shortest distance between the dendrite tip and the nearest branch point (or exit point from the soma). These measurements significantly underestimate the length of highly curved dendrites (< 10% of all dendrites); such dendrites were excluded from dendrite length and motility rate measurements.

Addition rate was obtained by dividing the total number of tips added (appearance of a tip from one time-point to the next) by the total duration of the time-lapse recording. The extension rate for each dendrite was calculated by dividing the length increase by the time required for the extension. Retraction rates were calculated in a similar manner. Dendrite lifetime was calculated by multiplying the number of time-points a dendritic tip was present by the interval between two time-points. Defined lifetimes could be assigned to tips that appeared and disappeared during the course of the entire time-lapse recording. However, for those dendrites that were either present at the first time-point or persisted at (or perhaps beyond) the last time-point, only minimum lifetimes could be assigned.

## Abbreviations

aPKC: atypical protein kinase C; CFP: cyan fluorescent protein; dpf: days post-fertilization; GCL: ganglion cell layer; GFP: green fluorescent protein; hpf: hours post-fertilization; ILM: inner limiting membrane; IPL: inner plexiform layer; OLM: outer limiting membrane; PBS: phosphate-buffered saline; RGC: retinal ganglion cell; wt: wild type; YFP: yellow fluorescent protein.

## Competing interests

The authors declare that they have no competing interests.

## Authors' contributions

J-HC carried out the experiments and analysis. C-BC and M-YL generated *isl2b:MGFP *transgenic fish, J-HC, BAL and ROLW designed the experiments, J-HC and ROLW wrote the manuscript. All authors read and approved the final manuscript.

## Supplementary Material

Additional file 1 **Time-lapse movie of RGC dendritic development *in vivo *(cell 1)**. Movie of the *isl2b:MGFP*-expressing cell in Figure 2. Images were acquired every 30 minutes to 2 hours between 41 and 47 hpf. Apical direction is upwards. Click here for file

Additional file 2 **Time-lapse movie of RGC dendritic development *in vivo *(cell 2)**. Example of cell with exuberant dendritic growth transitioning from an unbiased to a biased dendritic arbor. Cell was transiently expressing *ath5:gap-gfp*. Images were acquired commencing at 48 hpf at 10- to 40-minute intervals except for the final time-point, which was 2 hours after the previous time-point. Apical direction is upwards. Click here for file

Additional file 3 **Rotation of a RGC with an unbiased arbor**. Three-dimensional rotation of the cell with an unbiased arbor shown in Figure 3A (see Additional file 2 for the time-lapse movie of this cell). Dendritic tips are marked by red dots. The image was acquired at 51 hpf. Apical direction is upwards. Click here for file

Additional file 4 **Rotation of a RGC with a biased arbor**. Three-dimensional rotation of the maximum intensity projection of the cell with a biased arbor shown in Figure 3A. Dendritic tips are marked by red dots. The image was acquired at 52 hpf. Click here for file

Additional file 5 **Three-dimensional reconstruction of RGCs in the *has *retina (cell 1)**. Rotation of 4-dpf RGCs (*isl2b:MGFP*, center of image) with dendrites projecting in different directions in the *has *retina. A cell to the top right, adjacent to the outer limiting membrane, has a dendritic arbor oriented basally. Optical sections through the cells are also shown with BODIPY Texas Red staining. Neuropil-like regions are identified by the absence of cell bodies (dark holes). Axons are pseudo-colored in pink. Apical direction is towards the top. Movie scrolls deeper into the eye and back again. Click here for file

Additional file 6 **Three-dimensional reconstruction of a RGC projecting to neuropil at different locations in the *has *retina (cell 2)**. Rotation of the RGC (expressing *isl2b:MGFP *at 4 dpf), shown in Figure 7A, with dendrites projecting in three different directions in the *has *retina. The axon is pseudo-colored in pink. The series of optical sections through the cell are shown together with BODIPY Texas Red staining. Movie scrolls deeper into the back of the eye and back again. Apical direction is towards the right. Click here for file
